# Changes in white adipose tissue metabolism induced by resveratrol in rats

**DOI:** 10.1186/1743-7075-8-29

**Published:** 2011-05-10

**Authors:** Goiuri Alberdi, Víctor M Rodríguez, Jonatan Miranda, María T Macarulla, Noemí Arias, Cristina Andrés-Lacueva, María P Portillo

**Affiliations:** 1 Department of Nutrition and Food Science, Faculty of Pharmacy, University of País Vasco, Paseo de la Universidad 7, 01006 Vitoria, Spain; 2 RETICS RD06/0045/0003, Instituto de Salud Carlos III, Madrid, Spain; 3 Department of Nutrition and Food Science, XaRTA, INSA, Faculty of Pharmacy, University of Barcelona, Barcelona, Spain; 4 INGENIO-CONSOLIDER Program, Fun-c-food (CSD2007-063), Ministry of Science and Innovation, Barcelona, Spain

## Abstract

**Background:**

A remarkable range of biological functions have been ascribed to resveratrol. Recently, this polyphenol has been shown to have body fat lowering effects. The aim of the present study was to assess some of the potential underlying mechanisms of action which take place in adipose tissue.

**Methods:**

Sixteen male Sprague-Dawley rats were randomly divided into two groups: control and treated with 30 mg resveratrol/kg body weight/d. All rats were fed an obesogenic diet and after six weeks of treatment white adipose tissues were dissected. Lipoprotein lipase activity was assessed by fluorimetry, acetyl-CoA carboxylase by radiometry, and malic enzyme, glucose-6P-dehydrogenase and fatty acid synthase by spectrophotometry. Gene expression levels of acetyl-CoA carboxylase, fatty acid synthase, lipoprotein lipase, hormone-sensitive lipase, adipose triglyceride lipase, PPAR-gamma, SREBP-1c and perilipin were assessed by Real time RT-PCR. The amount of resveratrol metabolites in adipose tissue was measured by chromatography.

**Results:**

There was no difference in the final body weight of the rats; however, adipose tissues were significantly decreased in the resveratrol-treated group. Resveratrol reduced the activity of lipogenic enzymes, as well as that of heparin-releasable lipoprotein lipase. Moreover, a significant reduction was induced by this polyphenol in hormone-sensitive lipase mRNA levels. No significant changes were observed in other genes. Total amount of resveratrol metabolites in adipose tissue was 2.66 ± 0.55 nmol/g tissue.

**Conclusions:**

It can be proposed that the body fat-lowering effect of resveratrol is mediated, at least in part, by a reduction in fatty acid uptake from circulating triacylglycerols and also in *de novo *lipogenesis.

## Background

Overweight and obesity are a major public health concern because they are spreading throughout the world across all age barriers, afflicting not only adults but also many children and adolescents. Moreover, obesity is associated with several chronic diseases, such as diabetes, stroke and hypertension [1]. Considerable efforts are being made to identify and characterize novel naturally-occurring molecules which are orally active and safe and can be employed for obesity prevention, using a broad range of *in vivo *and *in vitro *methodologies. In this context, polyphenols make up one of the molecule groups most frequently studied in recent years.

Resveratrol (*trans*-3,5,4'-trihydroxystilbene) is a phytolaexin polyphenolic compound occurring naturally in various plants, including grapes, berries and peanuts, in response to stress, as a defence mechanism against fungal, viral, bacterial infections and damage from exposure to ultraviolet radiation [2]. Moreover, this compound is now available in tablets on the market.

A remarkable range of biological functions have been ascribed to this molecule. For example, it acts as a cancer chemoprevention agent, a powerful anti-inflammatory factor and an antioxidant [3,4]. Its cardiovascular properties, including inhibition of platelet aggregation and promotion of vasodilation, by enhancing the production of nitric oxide, have also been described [5].

More recently, resveratrol has been proposed as a potential anti-obesity compound. It seems to mimic the effects of energy restriction, thus leading to reduced body fat and improved insulin sensitivity [6-13].

Several mechanisms have been proposed to explain the body fat-lowering effect of resveratrol. It should be emphasized that a great deal of work has been developed in isolated adipocytes, thus limiting the extrapolation of the results to the *in vivo *situation. In this context, results coming from *in vitro *studies, performed in adipocyte types (3T3-L1 cells, pig adipocytes and human adipocytes) have shown that resveratrol increases apoptosis [14,15], decreases proliferation and differentiation of pre-adipocytes [16-18] and reduces lipogenesis [18]. Moreover, in *in vivo *and *ex vivo *experiments resveratrol has been shown to increase mitochondrial biogenesis, thus increasing fatty acid oxidation, and to enhance epinephrine-induced lipolysis [8,10,19].

Taking that into consideration, in the present study we aimed to gain more insight on mechanisms of action underlying the reduction in body fat induced by resveratrol, which take place in adipose tissue at the level of enzyme activities and gene expression.

## Methods

### Animals, diets and experimental design

The experiment was conducted with sixteen male Sprague-Dawley rats with an initial body weight of 180 ± 2 grams purchased from Harlan Ibérica (Barcelona, Spain) and took place in accordance with the institution's guide for the care and use of laboratory animals, with the approval of our internal animal ethics Committee (Reference protocol approval CUEID CEBA/30/2010), following European Community Council Directive. The rats were individually housed in polycarbonate metabolic cages (Techniplast Gazzada, Guguggiate, Italy) and placed in an air-conditioned room (22 ± 2°C) with a 12 h light-dark cycle. After a 6 d adaptation period, rats were randomly divided in 2 dietary groups of eight animal each (Control and Resveratrol) and fed on a commercial obesogenic diet, high in sucrose (20.0%) and fat (22.5%) (Harlan Iberica, TD.06415). Due to the fact that in a previous study from our laboratory we observed a body-fat lowering effect of resveratrol at a dose of 30 mg/kg/d [20], this dose was used in the present study. Resveratrol was added to the diet, as previously reported in the mentioned paper [20]. All animals had free access to food and water. Food intake and body weight were measured daily.

At the end of the experimental period (6 weeks) animals were sacrificed, under anaesthesia (chloral hydrate) by cardiac exsanguination, between 9.00 a 12.00 a.m. White adipose tissue from different anatomical locations (perirenal, epididymal, mesenteric and subcutaneous) was dissected, weighed and immediately frozen.

### Extraction and analysis of RNA and quantification by reverse transcription-polymerase chain reaction (RT-PCR)

Total RNA was isolated from 100 mg of epididymal adipose tissue using Trizol (Invitrogen, Carlsbad, California, USA), according to the manufacturer's instructions. RNA samples were then treated with DNA-free kit (Ambion, Applied Biosystems, Austin, Texas, USA) to remove any contamination with genomic DNA. The yield and quality of the RNA were assessed by measuring absorbance at 260, 270, 280 and 310 nm and by electrophoresis on 1.3% agarose gels. 1.5 μg of total RNA of each sample was reverse-transcribed to first-strand complementary DNA (cDNA) using iScript™ cDNA Synthesis Kit (Bio-Rad, Hercules, California, USA).

Relative acetyl-CoA carboxylase (ACC), fatty acid synthase (FAS), sterol-regulatory element binding protein-1c (SREBP-1c), adipose triglyceride lipase (ATGL), hormone sensitive lipase (HSL), Perilipin, LPL and peroxisome proliferator-activated receptor γ (PPARγ) mRNA levels were quantified using Real-time PCR with an iCycler™ - MyiQ™ Real time PCR Detection System (BioRad). β-actin mRNA levels were similarly measured and served as the reference gene.

Quantification of ACC, FAS, SREBP-1c, ATGL, Perilipin, LPL, PPARγ and β-actin by SYBR^® ^Green chemical Real time PCR: 0.1 μL of each cDNA were added to PCR reagent mixture, SYBR^® ^Green Master Mix (Applied Biosystems), with the sense and antisense primers (300 nM each). The PCR parameters were as follows: initial 2 min at 50°C, denaturation at 95°C for 10 min followed by 40 cycles of denaturation at 95°C for 15s, annealing for 30s and extension at 60°C for 30s. Information concerning specific sense/antisense primers and PCR annealing temperatures are described in Table 1.

**Table 1:** Primers and annealing temperatures for PCR amplification of each gene studied.

SYBR^® ^Green RT-PCR:			
Primers	Sense primer	Antisense primer	Annealing Temperature
ACC	5'-GGACCACTGCATGGAATGTTAA-3'	5'-TGAGTGACTGCCGAAACATCTC-3'	60.6°C
FAS	5'-AGC CCC TCA AGT GCA CAG TG-3'	5'-TGCCAATGTGTTTTCCCTGA-3'	60.0°C
SREBP-1c	5'-GCGGACGCAGTCTGGG-3'	5'-ATGAGCTGGAGCATGTCTTCAAA-3'	67.3°C
ATGL	5'-CACTTTAGCTCCAAGGATGA-3'	5'-TGGTTCAGTAGGCCATTCCT-3'	62.0°C
Perilipin	5'-AGAGGAGACAGATGAGGAGGAAG -3'	5'-AGATGGTGTTCTGCAGAGTCTTC -3'	63.9°C
LPL	5'- CAGCTGGGCCTAACTTTGAG -3'	5'- CCTCTCTGCAATCACACGAA -3'	61.5°C
PPARγ	5'-ATTCTGGCCCACCAACTTCGG -3'	5'-TGGAAGCCTGATGCTTTATCCCCA -3'	64.5°C
β-Actin	5'-ACGAGGCCCAGAGCAAGAG-3'	5'-GGTGTGGTGCCAGATCTTCTC-3'	60.0°C

ACC, acetyl-CoA carboxylase; FAS, fatty acid synthase; SREBP-1c, sterol-regulatory element binding protein-1c; ATGL, adipose triglyceride lipase; LPL, lipoprotein lipase; PPAR, peroxisome proliferator-activated receptor.

Quantification of HSL and β-actin by Taqman Real Time PCR: in the case of β-actin, 1 μL of each cDNA was added to PCR reagent mixture, Premix Ex TaqTM (Takara, Madison, Wisconsin, USA), with the sense (300 nM β-actin) and antisense primers (300 nM β-actin) and probe (0.5 μM β-actin). Specific primers were designed and synthesized commercially (Eurogentec, Liège, Belgium) and the sequences were as follows:

β-actin: 5'- TCT ATG AGG GCT ACG CTC TCC -3' (forward)

5'- CAC GCT CGG TCA GGA TCT TC -3' (reverse)

5'- FAM- CCT GCG TCT GGA CCT GGC TGG C -TAMRA-3' (probe)

For HSL mRNA levels 2 μL of each cDNA was added to PCR reagent mixture, TaqMan^® ^Universal PCR Master Mix, (Applied Biosystems), with 20X TaqMan^® ^Gene Expression Assay Mix containing specific primers and probes (Rn 00563444; Applied Biosystems).

The PCR parameters were as follows: denaturation at 95°C for 15s for β-actin and for 10 min for HSL, followed by 40 cycles of denaturation at 95°C for 5 s for β-actin and 15s for HSL and combined annealing and extension at 60°C for 30s for β-actin and 60s for HSL.

Gene expression analysis was performed using the Comparative threshold cycle (Ct) method [21]. Amplification of β-actin sequence was performed in parallel and was used to normalize values obtained for target genes.

### Enzyme activities

For total lipoprotein lipase (LPL) activity determination, homogenates of epididymal adipose tissue (250 mg) were prepared in 750 μL of 10 mM HEPES buffer (pH 7.5) containing 1 mM DTT, 1 mM EDTA, 250 mM sucrose and 2 g/L heparin and used as enzyme source. For heparin-releasable LPL (HR-LPL) activity determination, 250 mg of adipose tissue were incubated in 750 μL of 10 mM HEPES buffer (described above) at 37°C during 30 min.

Enzyme activity was assessed following the method described by Del Prado *et al*. [22], with modifications [23]. Total LPL and HR-LPL activities were calculated by subtracting non-LPL lipolytic activity in the presence of NaCl from the total lipolytic activity, determined without NaCl. Both total and HR-LPL activities were expressed as nmol oleate released per minute per gram of tissue.

For lipogenic enzyme analysis, 1 g of adipose tissue was homogenized in 5 mL of buffer (pH 7.6) containing 150 mM KCl, 1 mM MgCl_2_, 10 mM *N*-acetyl-cysteine and 0.5 mM dithiothreitol. After centrifugation at 100 000 *g *for 40 min at 4°C, the supernatant fraction was used for quantification of enzyme activities. Acetyl-CoA carboxylase (ACC, E.C. 6.4.1.2), fatty acid synthase (FAS, E.C. 2.3.1.85), glucose-6-phosphate dehydrogenase (G6PDH, E.C. 1.1.1.49) and malic enzyme (ME, E.C. 1.1.1.40) activities were measured as previously described [24]. Enzyme activities were expressed as follows: nmol NADPH consumed per minute per mg of protein for FAS, as nmol NADPH produced per minute per mg of protein for G6PDH and ME, and as nmol bicarbonate incorporated/min, per mg protein for ACC.

### Sample preparation and determination of resveratrol metabolites in adipose tissue

Analysis was carried out by (LC-MS/MS) after a solid-phase extraction (SPE), as described previously by Urpí-Sardà *et al*. and Andres-Lacueva *et al*. [25-27]. Liquid chromatography-tandem MS Liquid chromatography analyses were performed using an ACQUITY UPLC (Waters, Mildford, Massachusetts, USA) and a triple quadrupole mass spectrometer (API 3000) from Applied Biosystems (PE Sciex, Concord, ON, Canada), equipped with a Turbo IonSpray source operated in the negative-ion mode. For quantification of resveratrol metabolites in tissue samples, the multiple reaction monitoring (MRM) mode was used. All of them were quantified using a six-point calibration curve between 0.1 and 10 μg/g determined by weighted (1/x2) linear regression. The total amount of resveratrol metabolites was expressed as nanogram of *trans*-resveratrol equivalents per gram of tissue. The precision and accuracy of the method met the criteria of that validated by Urpí-Sardà *et al*. [26]. The Limit Of Detection (LOD) of the method was 0.045 μg/g and the Limit Of Quantitation (LOQ) was 0.1 μg/g.

#### Statistical analysis

Results are presented as means ± standard error of the means. Statistical analysis was performed using SPSS 17.0 (SPSS Inc. Chicago, Illinois, USA). Data were analysed by Student's *t *test. Statistical significance was set-up at the *P *< 0.05 level.

## Results

### Body weight, food intake and white adipose tissue weights

**Figure 1:**
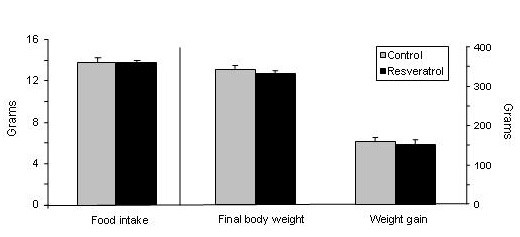
**Food intake, final body weight and body weigt gain**. Values are means for eight animals per group with the standard errors of the means, shown by vertical bars.

**Figure 2:**
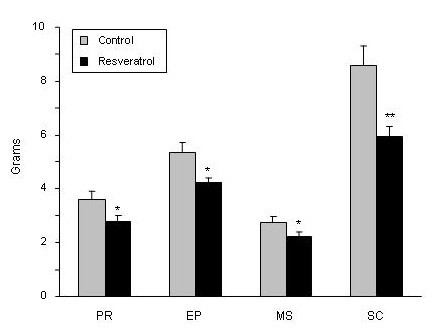
**White adipose tissue weights in experimental groups**. Values are means for eight animals per group with the standard errors of the means, shown by vertical bars. *P < 0.05; **P < 0.01. PR: perirenal; EP: epididymal; MS: mesenteric; SC: subcutaneous.

No statistical differences in food intake (*P *= 0.79), final body weight (*P *= 0.62) and body weight gain (*P *= 0.65) were found between experimental groups (Figure 1). Nevertheless, resveratrol significantly reduced adipose tissue (AT) weight in the four anatomical locations analysed (Figure 2). The magnitude of this reduction was as follows: 22.2% in perirenal AT, 21.2% in epididymal AT, 22.8% in mesenteric AT and 30.5% in subcutaneous AT.

### Enzyme activities in adipose tissue

**Figure 3:**
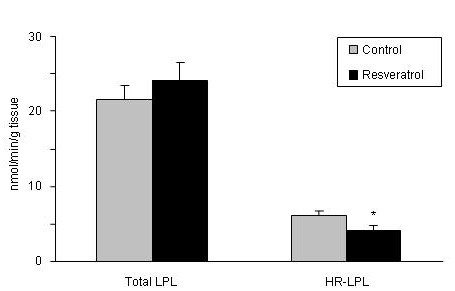
**Total and heparin-releasable (HR) lipopoprotein lipase activities in adipose tissue**. Values are means for eight animals per group with the standard errors of the means, shown by vertical bars. *P < 0.05.

**Figure 4:**
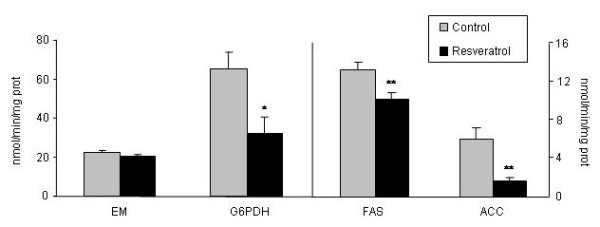
**Lipogenic enzyme activities in adipose tissue**. Values are means for eight animals per group with the standard errors of the means, shown by vertical bars. *P < 0.05; **P < 0.01. G6PDH: Glucose-6P-dehydrogenase; ME: malic enzyme; FAS: fatty acid synthase; ACC: acetyl-CoA-Carboxylase.

Only HR-LPL, the active fraction of the enzyme, was significantly reduced in resveratrol-treated rats (P < 0.05) (Figure 3). With regard to enzymes involved in *de novo *lipogenesis, rats fed the resveratrol-supplemented diet showed significantly reduced activities for G6PDH (*P *< 0.05), FAS (*P *< 0.01) and ACC (*P *< 0.01) (Figure 4).

### Enzyme and transcription factor expressions in adipose tissue

No significant changes were induced by resveratrol feeding in the expression of lipogenic enzymes (ACC and FAS) and LPL or the transcriptional factors regulating their activities, SREBP-1c and PPARγ. As far as lipases are concerned, although ATGL expression was not changed, in the case of HSL a significant decrease in Ct was observed, indicating that this lipase expression was raised by resveratrol. Finally, the expression of perilipin, a protein involved in the lipolytic process, was not modified (Table 2).

**Table 2:** mRNA levels in adipose tissue.

	Control	Resveratrol	
ACC	4.13 ± 0.57	4.02 ± 0.445	NS
FAS	1.91 ± 0.65	1.83 ± 0.74	NS
LPL	6.92 ± 0.43	7.00 ± 1.02	NS
SREBP-1c	3.23 ± 0.77	3.88 ± 0.69	NS
PPARγ	6.66 ± 0.49	6.56 ± 1.26	NS
HSL	4.65 ± 0.54^a^	1.99 ± 0.38^b^	*P <*0.05
ATGL	5.44 ± 0.42	5.64 ± 0.73	NS
Perilipin	1.73 ± 0.44	3.02 ± 0.60	NS

Values are given as Ct means ± standard errors of the mean (n = 8).

### Resveratrol and its metabolites in adipose tissue

*Trans*-resveratrol was not present in adipose tissue but several glucuronide and sulfate metabolites were found (i.e. *trans-*resveratrol-3-*O-*glucuronide, *trans*-resveratrol-3-sulfate, *trans- *resveratrol-4'-sulfate, *cis-*resveratrol-3-sulfate; *trans*-resveratrol-3,4-disulfate). The total amount of resveratrol metabolites found in adipose tissue from treated animals, expressed as resveratrol equivalents, was 2.66 ± 0.55 nmol/g tissue.

## Discussion

As explained in the introduction section, in recent years resveratrol has come to be a molecule of potential interest in reducing body fat. Nevertheless, information concerning the effects of resveratrol in animal models is rather scant.

In general terms, our results reporting a reduction in body fat in rats fed on a diet supplemented with resveratrol are in good accordance with those published by other authors [8,11-13]. It is important to point out that the effective dose in our experimental design, as well as those used by other authors, is far greater than the amount usually ingested by humans (100-930 μg/d) [28,29], meaning that the positive effects of this molecule on body fat would only be achieved by the intake of resveratrol pills or functional foods enriched with this molecule.

The amount of triacylglycerols accumulated in adipose tissue results from the balance among several metabolic pathways which take place in the tissue, such as *de novo *lipogenesis, fatty acid uptake from circulating triacylglycerols and lipid mobilization. In order to shed light onto the reasons that can explain the reduction in body fat achieved by resveratrol, the effects of this molecule on these pathways were assessed.

To evaluate the effect on fatty acid uptake from circulating triacylglycerols the activities of total LPL and heparin-releasable LPL (HR-LPL) were measured. As far as we know this is the first study which addresses the effects of resveratrol on this enzyme activity. Heparin-releasable LPL, the active form of this enzyme which is placed in adipose tissue endothelium, was significantly reduced by resveratrol. By contrast, when the expression of LPL and PPARγ, the transcriptional factor that regulates its activity, were measured no significant changes were observed. These data suggest that the effect of resveratrol on adipose tissue is mediated, at least in part, by a reduction in fatty acid uptake from triacylglycerols in circulating lipoproteins, and that the effect on LPL occurs at a post-transcriptional level. As far as we know this is the first study which addresses the effects of resveratrol on this enzyme activity.

With regard to *de novo *lipogenesis, in the present study a significant reduction was observed in the activity of FAS and G6PDH. The effects of resveratrol on G6PDH and FAS activities cannot be compared with other data in the literature because the effects of this polyphenol on these enzyme activities have not been addressed so far. The effect of resveratrol on ACC is in good accordance with the results previously reported by Rivera *et al*. in obese Zuker rats [11].

When we analysed the expressions of ACC and FAS we found that they were not modified by resveratrol, in line with the lack of change in SREBP-1c expression. Thus, our results suggest that, under the present experimental conditions, reduced lipogenesis contributes to the reduction in body fat induced by resveratrol, and that the effects on ACC and FAS occur, as well as in LPL, at a post-transcriptional level. It the case of ACC, it has been reported that the phosphorylation/desphosphorylation process regulated by AMPK plays a crucial role in the control of enzyme activity [30]. In this context, it is noteworthy that resveratrol has been reported to activate AMPK [11,12]. With regard to FAS, although one of the most important regulatory mechanisms for this enzyme takes place at the transcriptional level, other post-transcriptional mechanisms also contributes to enzyme activity regulation [31,32].

The effects of resveratrol on lipolysis have been analysed by Szkudelska *et al*. [33]. They observed enhanced lipolytic response to epinephrine induced by resveratrol. They suggested that this effect results, at least partially, from increased cAMP in adipocytes. Although it is well known that the main mechanism by which lipases are activated is phosphorylation, in order to gain more insight concerning the effects of resveratrol on this metabolic pathway, in the present study the expression of the two main adipose tissue lipases was measured. No changes were observed in mRNA levels of ATGL. By contrast, consistent with the previous demonstration of a positive effect of resveratrol on lipolysis [33], HSL expression was increased in resveratrol-treated animals, showing that this molecule acts differently on both lipases.

The effect of resveratrol on the expression of perilipin was also analysed. Resveratrol did not modify perilipin expression, suggesting that an effect on this protein does not underly the reduction in adipose tissue weight induced by resveratrol. Nevertheless, potential changes in perilipin phospohorylation cannot be discarded.

Some of the results related to the effects of resveratrol on gene expression in the present work are not in good accordance with some published studies performed in cultured cells. Thus, Rayalam *et al*. [14], reported that resveratrol repressed PPARγ, SREBP-1, FAS, LPL and HSL in 3T3-L1 adipocytes when these cells were incubated with 25 μM resveratrol. Floyd *et al*. [34] also found reduced expression of PPARγ and LPL in 3T3-L1 adipocytes treated with 50 μM resveratrol. The discrepancy between *in vitro *results and those reported in the present study is probably due to the fact that the resveratrol concentrations used with isolated cells, 25-50 μM, exceed the amount of this polyphenol usually found in blood after oral resveratrol administration (1.2-1.5 μM) [35,36], and consequently that reaching adipose tissue *in vivo*. In the present study *trans*-resveratrol was not found in adipose tissue due to its very short half life, but the total average amount of resveratrol metabolites in this tissue after treatment with resveratrol at a dose of 30 mg resveratrol/kg body weight/day was only 2.66 nmol/g. In addition, it is important to take into account that adipocytes are exposed to a great amount of changes in physiological stimuli under *in vivo *conditions, which are not considered in *in vitro *studies.

Thus, it should be emphasized that although some of the results obtained in *in vitro *studies by using isolated cells are quite promising and suggest the anti-obesity effect of resveratrol, it is important to be cautious and to check both the effects as well as the mechanisms of action, firstly in animal models and then in humans.

## Conclusion

Taken together, the results of the present work suggest that the body fat-lowering effect of resveratrol is mediated, at least in part, by a reduction in fatty acid uptake from circulating triacylglcyerols, as well as in *de novo *lipogenesis in adipose tissue. Nevertheless, some actions of this polyphenol on organs and tissues with a relevant role in triacylglycerol metabolism, such as liver and skeletal muscle, should also be considered. These should be further investigated in order to determine its whole body-fat mechanism of action.

## List of abbreviations

(ACC): Acetyl-CoA carboxylase; (AT): Adipose tissue; (ATGL): Adipose triglyceride lipase; (FAS): Fatty acid synthase; (G6PDH): Glucose-6-phosphate dehydrogenase; (HSL): Hormone sensitive lipase; (LPL): Lipoprotein lipase; (HR-LPL): Heparin-releasable LPL; (LOD): Limit of Detection; (LOC): Limit of Cuantification; (ME): Malic enzyme; (MRM): Multiple reaction monitoring; (PPARγ): Peroxisome proliferator-activated receptor γ; (SPE): Solid-phase extraction; (SREBP-1c): Sterol-regulatory element binding protein-1c.

## Competing interests

The authors declare that they have no competing interests.

## Authors' contributions

GA, VRM and JM carried out RT-PCR analysis. GA, MT and NA assessed enzyme activities, CAL evaluated resveratrol metabolites in adipose tissue. MT, VMR and MPP designed the experiment and MPP was responsible for results discussion and manuscript redaction. All authors read and approved the final manuscript.
